# Thresholds versus Anomaly Detection for Surveillance of Pneumonia and Influenza Mortality

**DOI:** 10.3201/eid2611.200706

**Published:** 2020-11

**Authors:** Timothy L. Wiemken, Ana Santos Rutschman, Samson L. Niemotka, Daniel Hoft

**Affiliations:** Saint Louis University, St. Louis, Missouri, USA

**Keywords:** data science, machine learning, time series, infection, seasonality, vaccine, immunization, viruses, respiratory infections, influenza, pneumonia, vaccine-preventable diseases, surveillance

## Abstract

Computational surveillance of pneumonia and influenza mortality in the United States using FluView uses epidemic thresholds to identify high mortality rates but is limited by statistical issues such as seasonality and autocorrelation. We used time series anomaly detection to improve recognition of high mortality rates. Results suggest that anomaly detection can complement mortality reporting.

Lower respiratory tract infections, including pneumonia and influenza (P&I), are the leading cause of infectious disease–related death worldwide (*1*). Annually, up to 95,000 persons might die from P&I in the United States alone (*2*). Ongoing surveillance of risk factors for influenza acquisition, incident influenza disease, and clinical outcomes of influenza infection are a global public health priority (*3*). Ensuring that public health professionals and the public at large are informed about the incidence and severity of disease in the community is an important benefit of these surveillance programs. To fulfill surveillance needs in the United States, the Centers for Disease Control and Prevention maintains FluView (*4*), a public-facing web interface providing detailed results of their influenza surveillance program. Reports maintained on FluView range from spatial analytics of influenza-like illness to virologic surveillance, virus characterization, hospitalization rates, and P&I mortality. Each report is useful for focused interventions and planning at a personal, local, state, regional, and national level.

Mortality reporting in FluView is a particularly critical public health endpoint for P&I because early interventions can lessen these catastrophic outcomes. Currently, mortality is monitored and reported as epidemic if the percentage of total deaths is above a value termed the epidemic threshold. This threshold is defined at a P&I death rate 1.645 SDs above the seasonal baseline mortality (*5*) as measured by the National Center for Health Statistics mortality surveillance system. These statistics are useful but limited in their ability to detect abnormally high death rates because they do not rigorously account for common statistical issues inherent in influenza surveillance data, such as within- and between-season seasonality and autocorrelation (*6*). Without accounting for the complex temporal fluctuations (seasonality) and nonindependence of period-to-period data points (autocorrelation), traditional statistical methodologies might provide spurious results, leading to inappropriate conclusions. Because an essential aspect of surveillance is ensuring that robust statistical methods are used to provide a valid view of the state of disease or outcome, the exploration of innovative methods for computational surveillance of P&I outcomes is warranted. The objective of our study was to evaluate the utility of a novel anomaly detection algorithm for P&I mortality surveillance.

## The Study

For our study, we obtained national P&I mortality data from FluView for a 350-week period ranging from week 40 of 2013 through week 24 of 2020. First, we recreated the current FluView P&I mortality plot, shading areas above the epidemic threshold to more easily delineate mortality rates higher than this limit. Next, we used Twitter’s time-series decomposition and the generalized extreme studentized deviate anomaly detection algorithm to identify anomalous P&I mortality rates (*7*,*8*). For anomaly detection, default α (0.05) and maximum anomalies (20%) were used as options. Anomaly plots identify anomalies using red dots. We analyzed data using R version 4.0.1 (R Foundation for Statistical Computing, https://www.r-project.org).

Using current epidemic threshold methodologies, we found that 72 (20.6%) of weekly P&I mortality rates were beyond the epidemic threshold (Figure, panel A). P&I mortality rates spiked above the epidemic threshold in approximately the same weeks every year since week 40 of 2013. Anomaly detection identified 17 (4.9%) P&I mortality rates as abnormally high (Figure, panel B). To ensure that this methodology can be continually used into the future, we also created a free, open-source, web-based application to recreate both figures on demand as data are updated (https://surveillance.shinyapps.io/fluview). Once loaded, the current national data are pulled from FluView and analyzed on the first tab. The anomaly plot and the updated current FluView P&I mortality surveillance plots are then displayed. For this web application, we included the options to modify some basic functionality of the anomaly detection algorithm with brief discussions of how they can be used (*7*,*8*). A second tab was created to enable upload of state-level P&I mortality data from FluView Interactive (https://gis.cdc.gov/grasp/fluview/mortality.html), providing the same anomaly detection plot.

**Figure:**
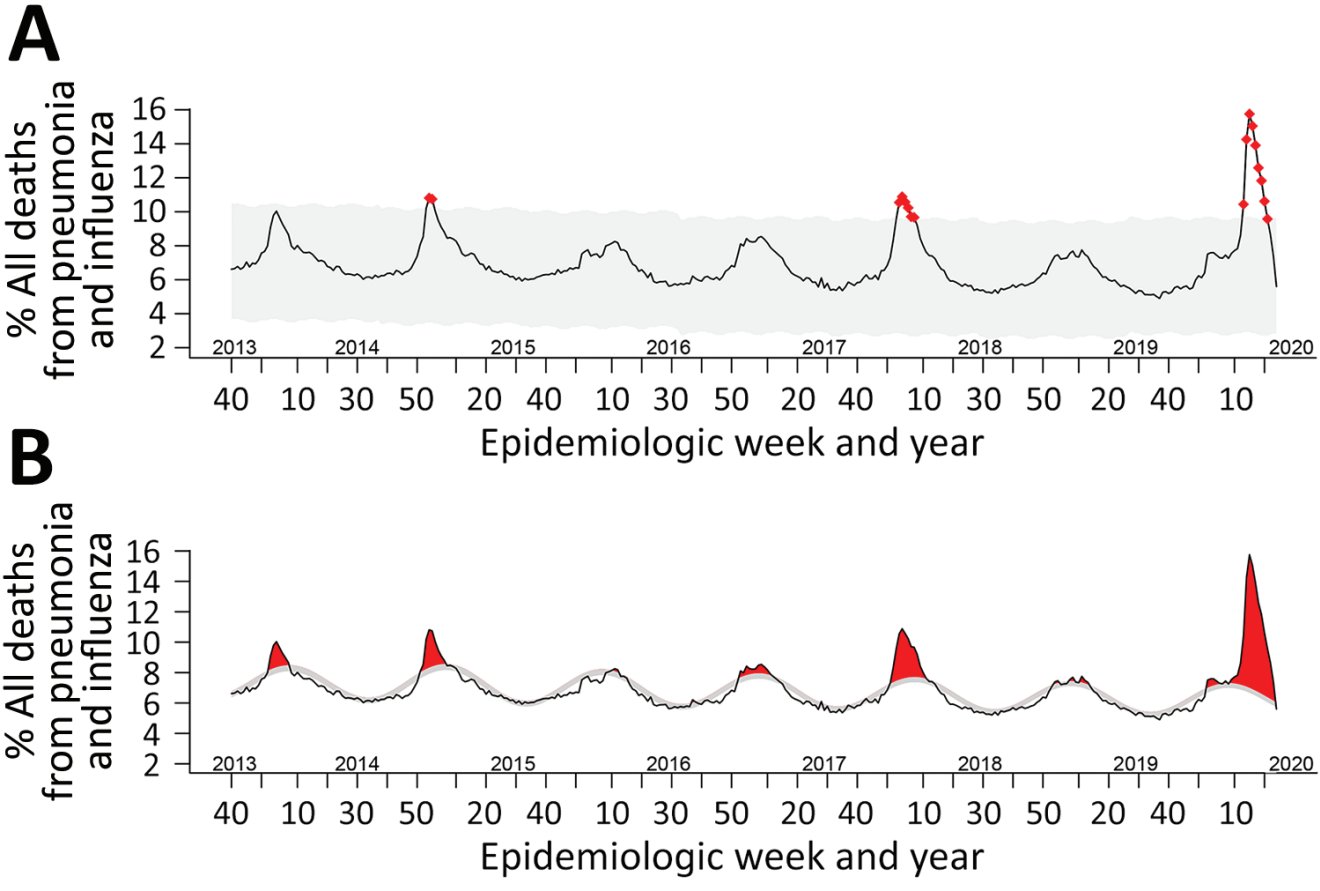
Pneumonia and influenza mortality surveillance using anomaly detection analysis versus threshold method, United States. A) Line chart representing anomaly detection analysis of surveillance. Red points indicate anomalous data points. B) Line chart representing the pneumonia and influenza mortality data using the standard FluView (*4*) threshold method. Gray areas indicate values between expected (baseline seasonal mortality) and the epidemic threshold. Red areas indicate areas beyond expected or epidemic threshold values.

## Conclusions

The current epidemic threshold for documenting P&I mortality in the United States cannot differentiate characteristic mortality rates during peak influenza season from unusually high mortality attributable to P&I. An important benefit of mortality surveillance is the identification of periods where rates are beyond a reasonable expectation such that adequate interventions can be developed to lower death rates in the community. Currently, P&I mortality rates are compared with a basic SD statistic obtained and averaged over seasonal baseline mortality estimates. This traditional approach does not account for seasonality or autocorrelative functions within and across influenza seasons (*6*). Given the advancements in computational power and the development of easy-to-interpret algorithms capable of filtering out these biases, alternative approaches for surveillance of P&I mortality at a national level should be considered to complement the current FluView methods. Our approach is one such alternative. Others such as the European EuroMoMo modeling (https://www.euromomo.eu) might also be applicable methods for bolstering our understanding of P&I mortality.

Although this particular anomaly detection might underestimate the frequency of abnormally high mortality rates, our approach is also likely to produce an additional, more focused message for public health professionals. Currently, P&I mortality peaks above the epidemic threshold at approximately the same time each year. Therefore, the existing approach might have a limited ability to provide public health professionals with the reports necessary to make informed interventions to limit mortality, such as through recalibrating targeted screening and preventative approaches, and to more accurately develop focused interventions such as vaccination campaigns. To accomplish this task, a computational method motivated by identifying outlying mortality rates should be used, with the caveat that mortality data must be reported in near real-time. Our approach provides such an outcome and might be useful for public health professionals in their quest to prevent and control P&I-related death. Our approach might also be useful for computational surveillance of other respiratory diseases, such as coronavirus.
